# Select intratumoral riboflavin-auxotrophic *Enterococcus* species enhance cell surface MR1 expression and MAIT TCR activation in lung cancer

**DOI:** 10.1073/pnas.2617943123

**Published:** 2026-08-18

**Authors:** Pakhi Birla, Lansaol Yang, Wanting Shan, Sakura Minamisawa, Omkar Dhaygude, Haritha Manoj, Alex J. Lee, Jacqueline Ferri, Ying Zheng, Andrew Northcutt, Hongni Fan, Hadley Beauregard, Zhen Zeng, Kellie N. Smith, Fyza Y. Shaikh, Cynthia L. Sears, Drew M. Pardoll, Franck Housseau

**Affiliations:** ^a^ Sidney Kimmel Comprehensive Cancer Center, Johns Hopkins University, Baltimore, MD 21205; ^b^Bloomberg ~ Kimmel Institute for Cancer Immunotherapy, Johns Hopkins School of Medicine, Baltimore, MD 21205; ^c^ Department of Pathology and Oncology, Faculty of Medicine, Juntendo University, Tokyo 113-842, Japan; ^d^ Department of Mechanical Engineering, Whiting School of Engineering, Johns Hopkins University, Baltimore, MD 21218; ^e^ Department of Medicine, Division of Infectious Disease, Johns Hopkins University, Baltimore, MD 21205

**Keywords:** MAIT, MR1, tumor microbiome, *Enterococcus*

## Abstract

Mucosal-associated invariant T (MAIT) cells are innate-like T cells capable of MR1-dependent immune surveillance, but how intratumoral bacteria modulate MR1 expression in human lung tumors remains unclear. We studied intratumoral MAIT cells from paired single-cell RNA and TCR sequencing datasets of tumor-infiltrating CD3 T cells isolated from non-small cell lung cancer tumors in patients receiving neoadjuvant PD-1 blockade therapy. MAIT cells were subclustered to identify conventional MAIT-associated TCR clonotypes, which were then used to examine how bacterial exposure impacts cell-surface MR1 expression and downstream MAIT TCR activation. We found that select intratumoral *Enterococcus* species (spp.) did not directly activate MAIT cells but enhanced MR1-dependent MAIT activation in the presence of exogenous 5-OP-RU by increasing cell-surface MR1 expression on antigen-presenting cells including dendritic cells, B cells, and mononuclear phagocytes. This increase in MR1 cell surface expression is modulated through a posttranscriptional mechanism consistent with altered intracellular processing and trafficking of MR1. These findings reveal a role for tumor-associated bacteria in modulating MR1-dependent innate-like T cell activation and provide a basis for future studies examining whether this process influences response to immune checkpoint blockade.

## Significance

MAIT cells are innate-like T cells that survey tissues through recognition of bacterially derived riboflavin metabolites presented by MHC class Irelated protein 1 (MR1), a nonclassical antigen-presenting molecule. Bacteria inside tumors may therefore shape these local immune responses, but the mechanisms are poorly understood. We show that select *Enterococcus* species found in lung tumors do not directly stimulate MAIT cells but instead increase MR1 surface expression and enhance MAIT cell activation through a posttranscriptional mechanism. These findings reveal a previously unrecognized role for tumor-associated bacteria in altering local immune recognition in cancer and may help inform future MR1-based immunotherapies.

The mucosal-associated invariant T (MAIT) cell subset, considered a nonconventional T cell subset, is a frequent innate immune population residing in mucosal sites, liver, and blood. MAIT cells can rapidly react to disruption of epithelium, mechanical and infectious, to restore mucosal homeostasis (1). This early response positions MAIT cells as a critical first line of defense against epithelial insults (2). MAIT cells are characterized by functional versatility allowing them to recognize and kill infected epithelial cells (3, 4) while also contributing to wound healing and tissue repair response (5). They are characterized by semi-invariant TCR alpha chains with a canonical gene recombination TCRAV1-2/J33, J12, or J20, and paired with a restricted set of beta chains (predominantly TCRB V6 or 20) (6). MAIT cells are part of a broader group of highly heterogeneous innate-like T cells which recognize target cells through the interaction of their TCR with a nonclassical MHC-I molecule, MHC-class-I-related protein 1 (MR1) (7). Their development in early-life and their functions at the epithelial barriers are mostly related to the regulation of their MR1 expression by bacterial ligands such as 5-(2-oxopropylideneamino)-6-D-ribitylaminouracil (5-OP-RU) and 5-(2-oxoethylideneamino)-6-D-ribitylaminouracil (5-OE-RU) derived from nonenzymatic reactions between bacterial riboflavin biosynthesis pathway intermediates 5-amino-6-D-ribitylaminouracil (5-A-RU) and methylglyoxal or glyoxal, respectively (8). The crosstalk between MAIT cells and bacteria is therefore an important contributor to the fitness of mucosal immunosurveillance (1, 2).

MAIT cells and other MR1 restricted T-cells can recognize a variety of unrelated bacteria and host-derived MR1 ligands associated with different cell types, tissues, and pathological contexts such as infection, cancer, and metabolism (9–16). Such ligands include cholic acid 7-sulfate (CA7S), derived from bile acid metabolism, which is recognized by _conv_MAIT(12), or tumor-associated nucleobase-derived compounds (e.g., carbonyl adduct of adenine called M3Ade) and pyridoxal/pyridoxal-5 phosphate (vitamin B6 derivative), which can trigger MR1 T cell activation (15, 17). Strikingly, healthy individuals and cancer patients possess MR1-restricted T cells which recognize cancer cells but not bacterially derived ligands (10).

The antitumor activity of these innate-like T cells is poorly understood, and their contribution to the clinical response of cancer patients to immunotherapy remains largely unknown. While MAIT cell function can be altered by PD-1 signaling and blockade of this immune checkpoint can restore their cytotoxic activity in vitro (18), analysis of single-cell transcriptomics and TCR sequencing of tumor-infiltrating lymphocytes (TIL) generated from patients with lung cancer receiving neoadjuvant anti-PD1 therapy showed that MAIT cells represented a sizeable proportion of T cells (19). We observed that one patient with a complete response exhibited the strongest MAIT cell clonal expansion.

Because MAIT cell abundance and function vary between individuals and can be influenced by microbial exposure (2), it is plausible that tumor-associated bacteria shape MAIT activity in patients treated with immune checkpoint blockade (ICB) (20, 21). Here, the crosstalk between bacteria and MAIT cells was examined to determine how intratumoral bacteria may modulate MR1-dependent activation of clonally expanded conventional MAIT cells. Select riboflavin-auxotrophic *Enterococcus* species enhanced cell-surface MR1 expression on antigen-presenting cells [APCs, e.g. B cells, dendritic cells (DC), and monocytic cell lines] and potentiated MAIT activation in the presence of exogenous 5-OP-RU. This effect was species-specific (*Enterococcus hirae*, *Enterococcus faecalis,* and *Enterococcus faecium* but not *Enterococcus avium*) and was not associated with increased MR1 transcription, supporting a posttranscriptional mechanism of MR1 regulation. These findings identify a previously unrecognized interaction between tumor-associated bacteria and MR1-dependent immune recognition in cancer patients.

## Results and Discussion

### Intratumoral Clonal Expansion of _conv_MAIT in Patients with Lung Cancer Treated with Neoadjuvant PD-1 Blockade.

**Fig. 1.**
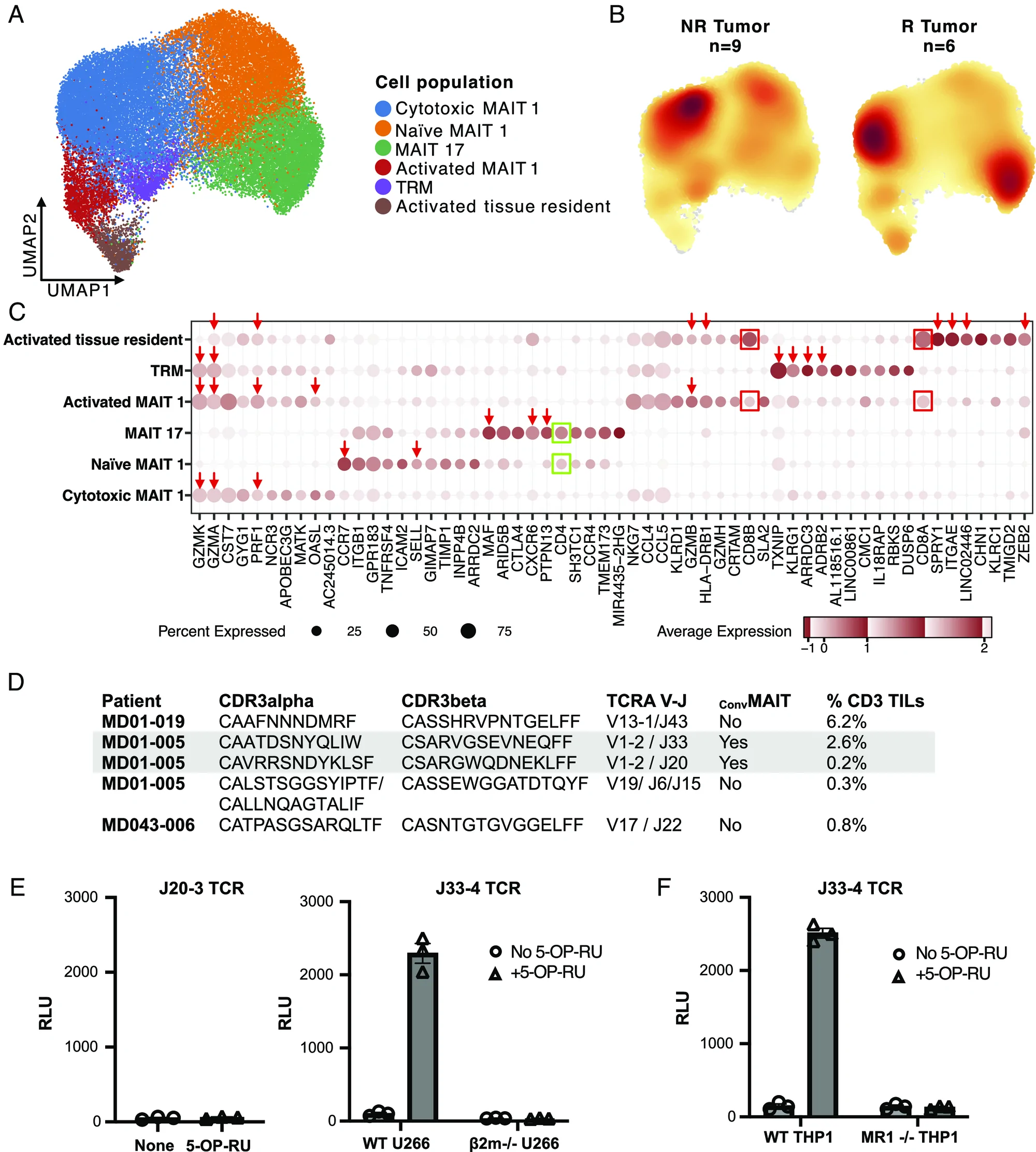
Single-cell RNA and TCR sequencing analysis of intratumoral MAIT cells in lung cancer patients. (*A*) UMAP representation of MAIT cell clusters resulting from the analysis of a published single-cell RNA sequencing dataset (19). (*B*) Cell density plots of MAIT cells in tumor tissue separated by patient response status (Responder, R, n = 6; Nonresponder, NR, n= 9). (*C*) Expression of top 10 markers for all 6 MAIT clusters. Red arrows indicate gene ID driving the cluster annotations. Red boxes indicate *CD8A* and *CD8B* genes, and green boxes indicate *CD4* gene. (*D*) Top 5 most frequent _conv_MAIT clonotypes among the CD3+ TILs. The table indicates the frequency of the _conv_MAIT clonotypes among CD3+ T cells, the CDR3α and CDR3β sequences, the nature of the canonical recombination (TCRAV1-2/J12,20,33), and the MAIT cluster (Fig. 1*A*) to which they belong. (*E*) *Left,* MD01-005 J20-3 TCR from predicted _conv_MAIT cell did not recognize MR1 in the TCR capture assay in the presence of 5-OP-RU; *Right*, MD01-005 J33-4 TCR from predicted _conv_MAIT cell recognized human myeloma cell line U266 but not β2m-KO U266 cells in the presence of 5-OP-RU. (*F*) MD01-005 J33-4 TCR from predicted _conv_MAIT also recognized the human phagocytic cell line, THP1, but not MR1-KO THP1 in the presence of 5-OP-RU.

To identify innate immune cell populations in the lung tumor microenvironment (TME), we analyzed previously published single-cell transcriptomic dataset from patient-matched tumor and distant normal lung tissues (19). The initial CD3+ T cell clustering identified a MAIT cell cluster representing **6.3**% of all T cells collected from 16 patients [7 pathological responses (R) and 9 without pathologic responses (NR)] (19). Across all tissue infiltrating T-cells analyzed, MAIT cells represented 9% in normal lung (n = 12) and 9% in tumor lung (n = 15). MAIT cells represented 8.6% of tumor-infiltrating T cells in R patients and 9.6% in NR patients. We further subclustered MAIT-annotated cluster into CD4negCD8neg cytotoxic MAIT1 (*PRF1^pos^, IFNGR1^pos^*), CD4pos Naïve MAIT1 (*CD62L^pos^, CCR7^pos^, SOCS1^pos^*), CD4pos MAIT17 (*MAF^pos^, CCR6^pos^, BATF^pos^, RORA^pos^, AHR^pos^, PTPN13^pos^*), CD8pos activated MAIT1 (*HLA-DRB1^pos^, PRF1^pos^, GZMA^pos^, GZMB^pos^*), tissue resident memory (TRM) MAIT1 (CD69*^pos^*, CD62L*^pos^*, IL7R*^pos^*, KLRG1*^pos^*, TXNIP*^pos^*, ADBR2*^pos^*, ARRDC3*^pos^*, STAT1*^pos^*, SOCS1*^pos^*, IFNGR1*^pos^*) and activated CD8pos TRM MAIT cells (*HLA-DRB1^pos^*, ITGAE*^pos^*, LINC02446*^pos^*, *SPRY1^pos^, ZEB2^pos^, PRF1^pos^, GZMA^pos^, GZMB^pos^*) (Fig. 1 *A* and *C* and *SI Appendix*, Table S1 contains top 20 differentially expressed genes by each cluster). Notably, we observed different densities of cells within the *cytotoxic MAIT1* cluster between R and NR, as well as a higher density of cells in the *MAIT17* and *activated tissue resident MAIT* clusters in responding tumors (Fig. 1*B*). Furthermore, while the c*ytotoxic MAIT1* cluster in the tumor of R patients had a significantly higher expression of cytotoxicity associated genes such as PRF1, NCR3, and NKG7 (*SI Appendix*, Fig. S1*A*), the same MAIT cluster in NR tumors was characterized by higher expression of the immunosuppressive TGFB activation pathway (*TGFB* and *ITGVA*) (22). These results suggest that the transcriptional profile of MAIT cells could be reflective of the tumor response to ICB. We herein paid attention to how the intratumoral bacteria and their metabolites, biosynthetic precursors of riboflavin (VitB2), can impact _conv_MAIT cell activation.

We then identified TCRVA1-2^pos^_conv_MAIT clonotypes from patient-matched single-cell RNA sequencing/single-cell TCR sequencing dataset analysis (1) (Fig. 1*D*) and selected the top 5 predominant tumor-infiltrating _conv_MAIT clonotypes to test MR1 recognition. TCRVA1-2^pos^_conv_MAIT clonotypes represented 7.5% of MAITs. The two most frequent _conv_MAIT TCR clonotypes, *MAIT17* MD01-005 J20-3 (TCRAV1-2/J20; 0.2% of the TIL) and *cytotoxic MAIT1* MD01-005 J33-4 (TCRAV1-2/J33; 2.6% of the TIL), were identified in R patient MD01-005 (Fig. 1*D*). Although the overall proportion of MAIT cells among T cells was similar between the R and NR patients, we found an increase of _conv_MAIT cells in R patients (16.7% v. 0.6% among MAIT cells in TILs of R and NR patients, respectively, and representing 1.44% v. 0.06% among all T cells). However, this increase was primarily driven by the remarkable expansion of J33-4 MAIT clonotype in patient MD01-005.

Because MAIT-annotated transcriptional clusters may include cells that are not functionally MR1-reactive, all mechanistic experiments in this study were subsequently focused on functionally validated _conv_MAIT clonotypes. MAIT TCR MD01-005 J20-3 and J33-4 TCRs were therefore cloned and validated for the MR1-dependent recognition of APCs in presence of the riboflavin biosynthesis-associated metabolite, 5-OP-RU (Fig. 1 *E* and *F*). Our results showed that clonotypes MD01-005 J33-4 but not J20-3 MAIT TCR recognized 5-OP-RU-induced MR1 on the human myeloma cell line U266, reinforcing the need to verify the MR1 dependence of T cell recognition, to functionally validate MAIT cell properties (Fig. 1*E*). We confirmed that MAIT TCR J33-4 recognized the human leukemia cell line THP1 but only in the presence of 5-OP-RU and MR1 (Fig. 1*F*). These data confirmed that patient MD01-005 J33-4 MAIT TCR activation is MR1 ligand dependent. The most abundant _conv_MAIT TCR clonotype, MD01-005 J33-4, overlapping with *cytotoxic MAIT1* cluster was therefore chosen to further probe how intratumoral bacteria impact the expression of MR1 on APC.

### Detection of Intratumoral Bacteria in Lung Cancer Patients and Assessment of Their _conv_MAIT TCR Activation Ability.

**Fig. 2.**
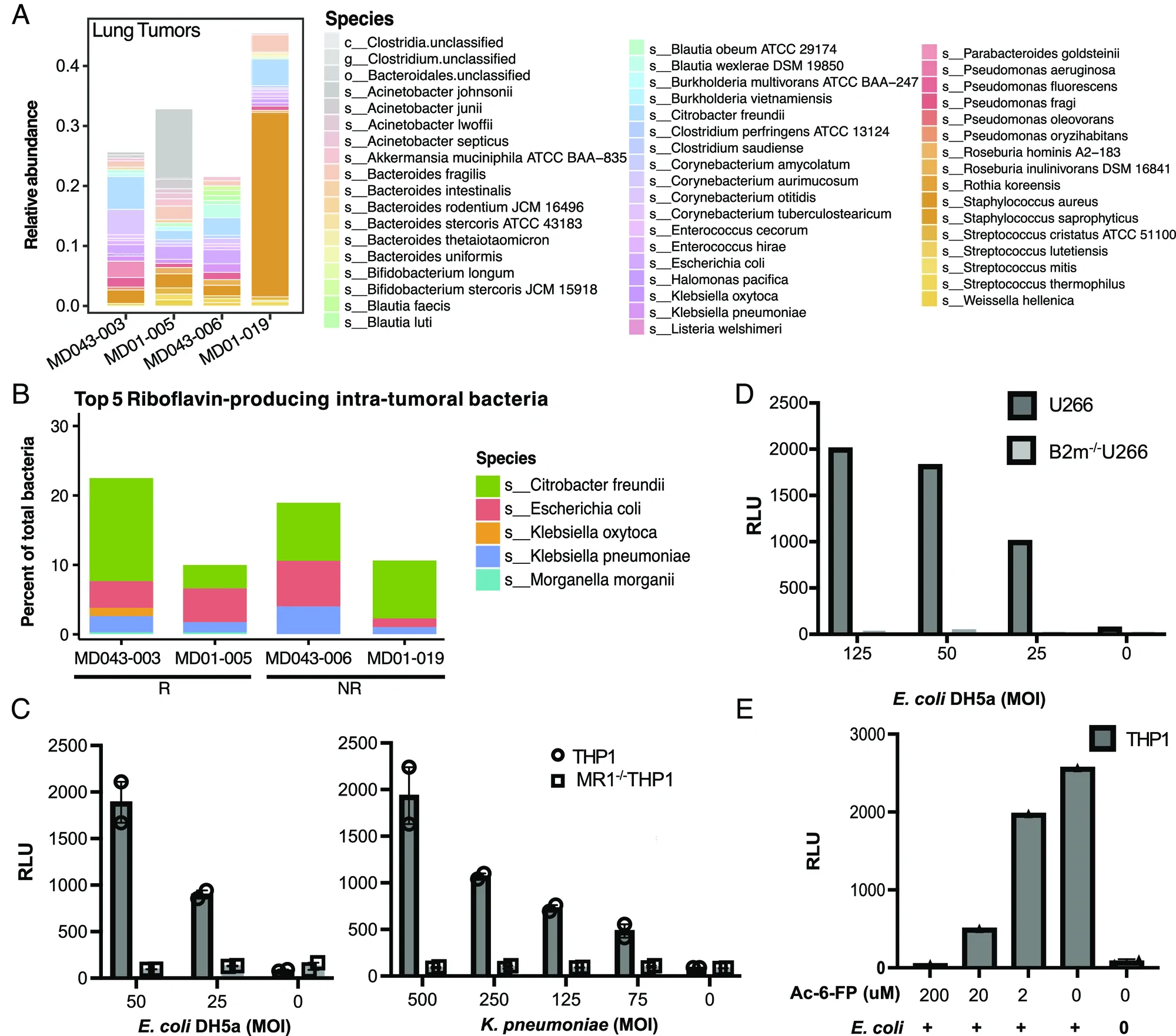
Intratumoral bacteria and riboflavin metabolism detection in lung cancer. (*A*) Relative abundance of bacteria species identified by 16S V3-V4 rRNA amplicon sequencing performed on DNA extracted from FFPE tumor tissue sections from four patients (MD01-019, MD01-005, MD043-006, and MD043-003). (*B*) proportion (out of total bacteria) of the top five 5-A-RU-producing intratumoral bacteria (*SI Appendix*, Fig. S1) per patient after contaminant bacteria removal. A literature-based pipeline was developed to predict the bacteria which will express at least the three required genes for 5-A-RU production (*SI Appendix*, Fig. S1*B*). (*C*) Recognition of WT and MR1^−/−^ THP1 by MD01-005 J33-4 MAIT TCR expressing Jurkat reporter cells in the presence of PFA-fixed *Escherichia coli* DH5α or *Klebsiella pneumoniae* at different multiplicities of infection (MOI). Experiments were performed in replicates (n = 2) and are shown as mean ± SEM. (*D*) Recognition of WT and b2m^−/−^ U266 by MD01-005 J33-4 MAIT TCR in the presence of PFA-fixed *E. coli* DH5α. (*E*) Inhibition of MD01-005 J33-4 MAIT TCR recognition of *E. coli* DH5α incubated THP1 by escalating Ac-6-FP doses (2, 20, 200 μM).

We performed 16S rRNA amplicon sequencing to profile intratumoral bacteria in a limited set (n = 4) of formalin-fixed paraffin embedded (FFPE) tumor samples and identified candidate species potentially capable of modulating MAIT activation (Fig. 2*A*). While we did not have fresh tumor tissue available for microbiological analysis, we used clinical and ATCC available strains of species identified by 16S rRNA amplicon sequencing to test their impact on MR1 expression and MAIT cell activation.

Next, to score how many of these bacteria had the ability to produce riboflavin-derived metabolite, 5-A-RU, we developed a literature-based meta-analysis pipeline predicting MR1-binding metabolite biosynthesis ability of the tumor microbiome (*SI Appendix*, Fig. S1 *B* and *C*). We included a total of three genes core for 5-A-RU production: *ribA*, *ribE,* and *yigB* (*PYRP2*)(*SI Appendix*, Fig. S1*B*) (2). We found that an average of 11% of the intratumoral bacteria in the 4 patients tested are reported to express the minimal three gene core required for 5-A-RU synthesis (Fig. 2*B* and *SI Appendix*, Fig. S1*C*). However, there was no association between predicted 5-A-RU biosynthesis and abundance of MAIT cells detected in our datasets (*SI Appendix*, Fig. S1*D*). Noteworthy, we found that several strains of bacterial species previously reported to produce riboflavin like *Bacteroides thetaiotaomicron* and *Staphylococcus epidermidis* did not induce herein MD01-005 J33-4 MAIT TCR activation demonstrating that bacterial strains can likely vary in their ability to promote MAIT activation (*SI Appendix*, Fig. S1*E*).

We then confirmed that MD01-005 J33-4 TCR recognized riboflavin-producing but not riboflavin-deficient bacteria in an MR1-dependent manner using paraformaldehyde (PFA) fixed *E. coli* and *K. pneumoniae* (both riboflavin synthesis proficient) vs. riboflavin-auxotrophic *E. hirae*. We showed that reporter Jurkat cells expressing MD01-005 J33-4 TCR are activated by wild type (WT) THP1 or myeloma U266 cell lines, but not by their MR1 deficient counterparts (MR1^−/−^ THP1, B2m^−/−^ U266) in the presence of *E. coli* or *K. pneumoniae* (Fig. 2 *C* and *D*). Finally, we saw that MD01-005 J33-4 MAIT TCR activation is inhibited by escalating concentrations of the MR1 antagonistic ligand Acetyl 6 formylpterin (Ac-6-FP) (Fig. 2*E*), which, although induces cell membrane expression of MR1 (*SI Appendix*, Fig. S1*F*) is not recognized by MAIT TCR (Fig. 2*E*) (23).

Altogether, although we were unable to isolate the patient-specific clinical bacterial strains, our findings using acquired strains suggested that identification of actual intratumoral bacteria as well as validation of the presence of the genes necessary to produce MR1 ligands would not be sufficient to accurately predict MAIT TCR recognition. This may be, in part, because expression of genes encoding enzymes driving riboflavin precursor biosynthesis is tightly regulated by other environmental and contextual factors (24). Furthermore, other metabolites such as folate can bind MR1 and block the activation of MAIT cells by riboflavin precursors (23, 25).

### Riboflavin-Auxotrophic *Enterococcus* spp., but not *E. coli*, Synergizes with 5-OP-RU, to Promote MR1 Expression on APC, Independently of Pathogen Associated Membrane Pattern (PAMP) Recognition.

**Fig. 3.**
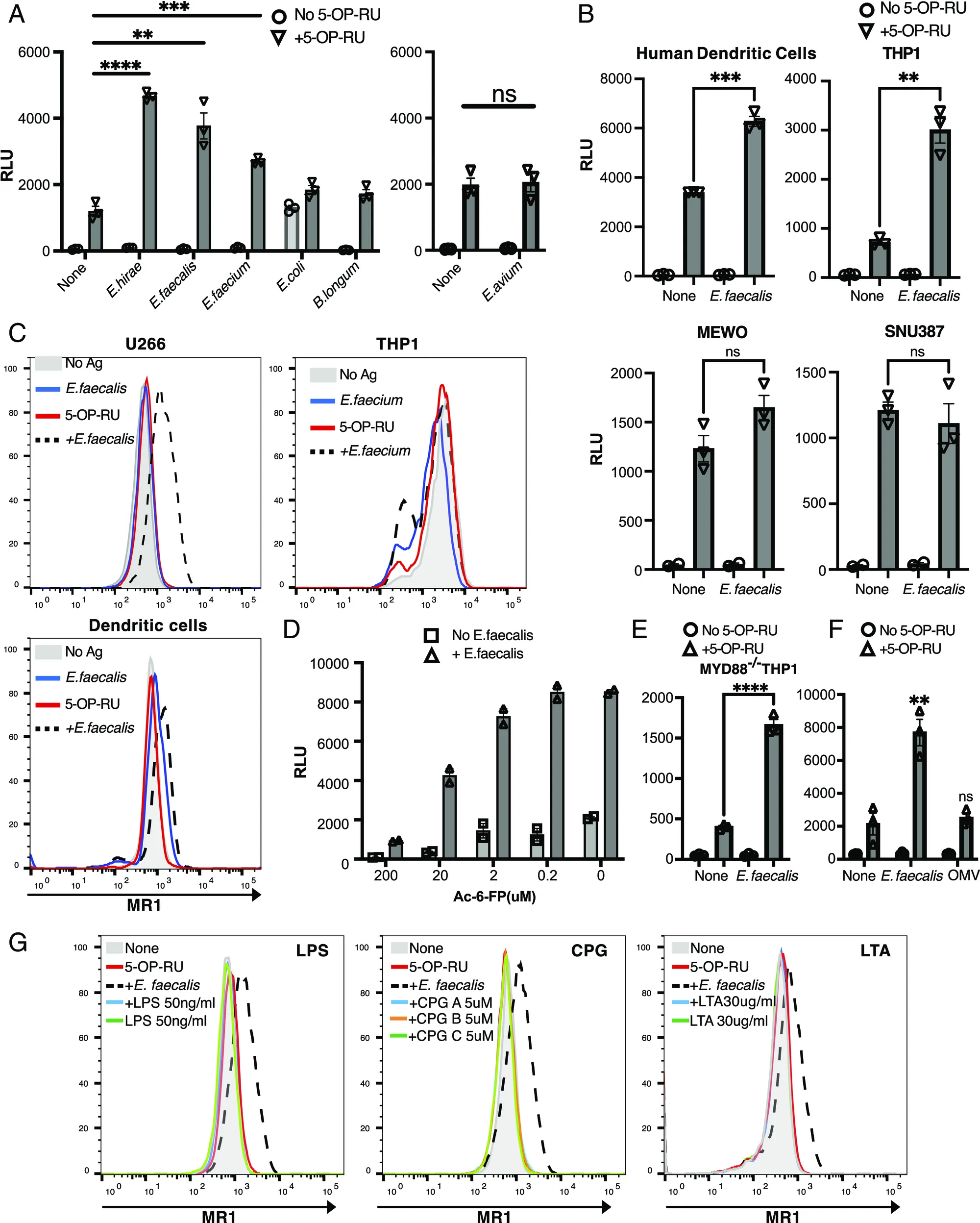
*Enterococcus* species synergize with 5-OP-RU to enhance MR1-dependent MAIT TCR activation. (*A*) J33-4 MAIT TCR recognition of U266 cells incubated overnight with (inverse triangles) or without (circles) 5-OP-RU, in the presence or absence of *E. hirae*, *E. faecalis*, *E. faecium*, *E. coli,* and *B. longum* (*Left*) and *E. avium* (*Right*, independent experiment). TCR activation is measured by bioluminescence reading. (*B*) Different APC (human DC, THP1, melanoma MeWO, and hepatocellular carcinoma SNU387) were tested for the synergistic effect of *E. faecalis* on activation of conventional MAIT TCR MD01-005 J33-4 in the presence (inverse triangles) or absence (circles) of 5-OP-RU. (*C*) Flow cytometric analysis of MR1 surface membrane expression in U266, THP1, and DC incubated overnight with or without 5-OP-RU in the presence or absence of *E. faecalis* or *E. faecium*. (*D*) Inhibition by Ac-6-FP (escalating doses from 0.2 to 200 μM) of recognition by MAIT TCR MD01-005 J33-4 of MR1 expressed on U266 cultured with or without 5-OP-RU (6.25 μM) in the presence (triangles) or absence (squares) of *E. faecalis* (MOI:125). (*E*) MAIT TCR J33-4 activation by MYD88^−/−^ THP1 cells incubated overnight with (triangles) or without (circles) 5-OP-RU (6.25 μM), in the presence or absence of *E. faecalis* (MOI:125). (*F*) MAIT TCR MD01-005 J33-4 activation by U266 cells incubated overnight with (triangles) or without (circles) 5-OP-RU (6.25 μM), in the presence or absence of *E. faecalis* (MOI:125) and *E. faecalis* derived outer membrane vesicles (OMV). (*G*) Flow cytometric analysis of MR1 surface membrane expression using the 26.5 anti-MR1 antibody staining of U266 cells incubated overnight in combination with *E. faecalis* (MOI:125), or LPS (TLR4 ligand; 50 ng/mL; *Left*), or CPG (TLR9 ligand; 5 μM, *Middle*) or LTA (TLR2 ligand; 30 μg/mL; *Right*). All data are shown as mean ± SEM with technical replicates represented by number of symbols in each graph. ****P < 0.0001, ***0.0001 < *P* < 0.001, **0.001 < *P* < 0.01, *0.01 < *P* < 0.05, ns (nonsignificant) by the unpaired *t* test (two-tailed). Each experiment was repeated at least two times. The representative experiment is shown.

Because bacteria can also produce antagonistic MR1 ligands (26), we further tested whether *E. hirae* can suppress MAIT activation. Unexpectedly, rather than inhibiting MAIT TCR recognition, *E. hirae* triggered a synergistic effect on MAIT activation with 5-OP-RU, compared to 5-OP-RU alone (Fig. 3*A*). Additionally, we found that both *E. faecalis and E. faecium* but not *E. avium,* exhibited the same synergistic effect. We confirmed that all the *Enterococcus* strains used herein were riboflavin auxotrophic by their genomic sequencing and metabolomic analysis (*SI Appendix*, Fig. S2 *A* and *B*). We then showed that the synergistic properties of *Enterococcus* spp. were not reproduced with other riboflavin-auxotrophic bacteria (*Bifidobacterium longum*) and were selective to professional APCs, including DC, monocytic (THP1), and B cell–derived cell line (human myeloma cell line U266) (Fig. 3*B*). Human Melanoma MeWo and hepatocellular carcinoma (HCC) SNU387 cell lines, although capable of activating MAIT cells in presence of 5-OP-RU, did not exhibit higher MAIT activating properties in presence of *Enterococcus* (Fig. 3*B*). We then verified the cell surface expression of bioactive refolded MR1 using the 26.5 antibody clone (27). While surface expression of MR1 was previously reported as transient and low, Fig. 3*C* shows that the surface expression of MR1 did not always correlate with TCR activation. In myeloma U266 cell line and human DCs, higher activation of MAIT TCR by the combination 5-OP-RU/*E. faecalis* paralleled the increased MR1 expression, but not in THP1 for which MR1 was not detectable (Fig. 3 *A* and *C*). Suppression of synergistic activation of MD01-005 J33-4 TCR expressing Jurkat cells by increasing concentrations of antagonistic ligand, Ac-6-FP, provided direct evidence of the role of increased MR1 cell surface expression in the synergy of MAIT TCR activation by *Enterococcus* (Fig. 3*D*).

Culture of U266 with both 5-OP-RU and *Enterococcus* did not increase the production of IL1β, IL-18 or IFNα in the supernatants compared to 5-OP-RU alone (*SI Appendix*, Fig. S2*D*). Furthermore, the synergistic effect was observed only with live or PFA-fixed *Enterococcus* spp.; neither lysed nor sonicated bacteria triggered synergy (*SI Appendix*, Fig. S2*E*). We therefore tested the possibility that *Enterococcus* spp. acted via PAMPs/pattern recognition receptor (PRR) interactions. However, Myd88-KO THP1 cells were as efficient as WT THP1 in activating MD01-005 J33-4 expressing Jurkat cells, (Fig. 3*E*). Finally, neither nonreplicative *E. faecalis*-derived OMV (Fig. 3*F*), nor TLR ligands phenocopied the synergistic effect of *Enterococcus* spp. (Fig. 3*G*). The species-specific nature of this effect, together with the lack of dependence on MyD88 signaling and failure of TLR ligands or outer membrane vesicles (OMV) to recapitulate the phenotype, argues against a PAMP-driven mechanism. Interestingly, the synergistic effect of *Enterococcus* spp. was observed with 5-OP-RU but not with PFA-fixed *E. coli*, suggesting that the source and form of the MR1 ligand (soluble vs. bacteria-associated) influence the ability of *Enterococcus* spp. to enhance MR1 expression and MAIT activation (*SI Appendix*, Fig. S2*F*).

### The Synergistic Effect of *Enterococcus* spp. on MR1-dependent MAIT Cell Activation Is Posttranscriptionally Regulated.

The expression of MR1 and riboflavin precursors is common, paradoxically also key for the selective activation of MAIT cells (28). Previous studies showed that the expression of MR1 is highly contextual and diversely regulated according to the cell types (reviewed in refs. 1 and 29).

**Fig. 4.**
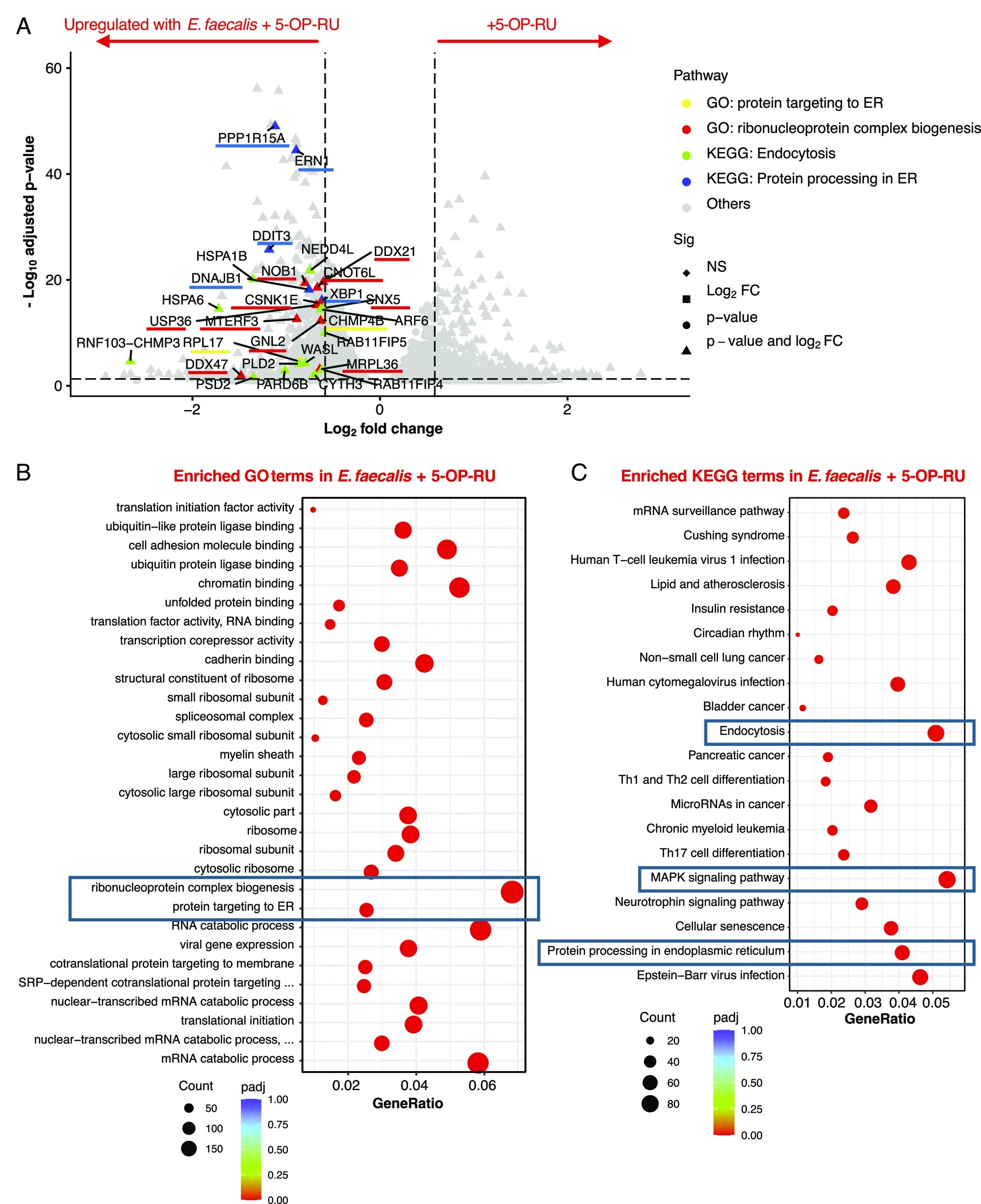
Differential effect of *E. faecalis* and *E. coli* on gene expression by human myeloma U266 cells in the presence or absence of 5-OP-RU. (*A*) volcano plot representing RNAseq analysis of U266 cells incubated with 5-OP-RU + *E. faecalis compared* to 5-OP-RU alone (adjusted *P*-value < 0.01 and LFC | > 1.5). Genes belonging to the GO signature *protein targeted to ER* and *ribonucleoprotein complex biogenesis* are annotated in yellow and red, respectively. Genes included in the KEGG signature *Protein processing in ER* and *Endocytosis* are annotated in blue and green respectively. (*B* and *C*) Dot plot visualization of the enriched GO (*B*) and KEGG (*C*) terms in 5-OP-RU + *E. faecalis* condition after RNAseq analysis of U266 cells cultured with 5-OP-RU + *E. faecalis* compared to 5-OP-RU alone.

To understand the effect of *Enterococcus* infection on MR1 upregulation, we next performed bulk RNA sequencing of U266 cells incubated in the presence or absence of 5-OP-RU with *E. faecalis* (riboflavin auxotrophic) vs. riboflavin-producing-*E. coli* (grp1 = U266; grp2 = U266/5-OP-RU; grp3 = U266/Ef; grp4 = U266/*E. coli*; grp5 = U266/5-OP-RU + Ef; grp6 = U266/5-OP-RU + *E. coli* Fig. 4). In contrast to upregulation of surface MR1 in the presence of 5-OP-RU and *E. faecalis* as compared to 5-OP-RU alone (Fig. 3*C*), *MR1* mRNA expression was not affected by *E. faecalis*, suggesting that *E. faecalis* enhances MR1 surface expression through a posttranscriptional mechanism. We also observed that incubation of U266 with the combination 5-OP-RU/*E. faecalis* was associated with the upregulation of GO terms *protein targeting to ER* and KEGG signatures *endocytosis*, *protein processing in ER* compared to 5-OP-RU alone (Fig. 4 *B* and *C*). Finally, compared with *E. coli*, *E. faecalis* coculture led to the upregulation of a set of gene expression linked to autophagy and lipophagy such as *DEPP1* (30), *MT1G* (31), *BBS12* (32), *FICD* (33), and *HILPDA* (34) (*SI Appendix*, Fig. S3).

Altogether, these transcriptomic data support a posttranscriptional regulation of MR1 expression consistent with *Enterococcus*-mediated alteration of MR1 intracellular trafficking.

### Synergistic Activity of *Enterococcus* on MR1 Expression Is Associated with Alteration of MR1 Intracellular Trafficking.

**Fig. 5.**
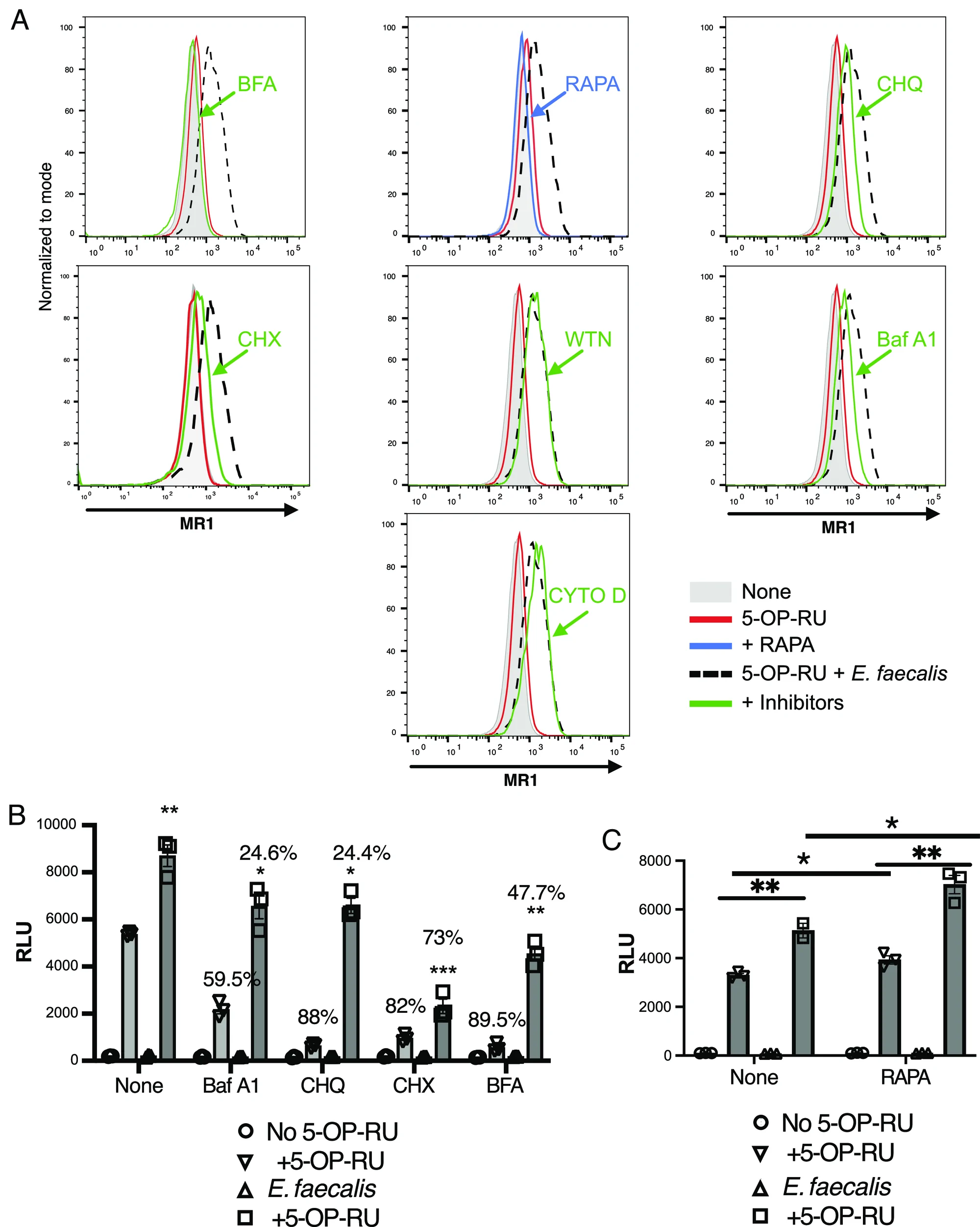
Pharmacological perturbation suggests intracellular regulation of surface MR1 expression by *Enterococcus* spp. (*A*) Flow cytometric analysis of MR1 expression by U266 using the 26.5 antibody clone after overnight incubation with 5-OP-RU and *E. faecalis* in the presence of Baf A1 (100 nM), ChQ (100 μM), CHX (20 μg/mL), BFA (3 μg/mL), and RAPA (100 nM). (*B* and *C*) 6 h-MR1 recognition assay using J33-4 MAIT TCR expressing Jurkat reporter cells and, as APC, U266 cells treated overnight with the drugs described in *A* (APCs were washed after incubation with pharmacological agents), %inhibition in each drug treated condition is shown above bars. Results are shown as relative values of TCR/NFAT-induced bioluminescence (RLU). All data are shown as mean ± SEM with technical replicates represented by number of symbols in each graph. ****P < 0.0001, ***0.0001 < *P* < 0.001, **0.001 < *P* < 0.01, *0.01 < *P* < 0.05, ns (nonsignificant) by the unpaired *t* test (two-tailed).

*E. faecalis* is capable of persisting in the endo-lysosomal compartment and resisting autophagy in macrophages via inhibition of the fusion of lysosome with late endosomes or autophagosomes, respectively (35, 36). We therefore tested whether intracellular regulatory pathways contribute to modulation of surface MR1 expression. Under conditions optimized to preserve cell viability (>80%), we used a set of pharmacological agents which induce (Rapamycin; RAPA) or inhibit (*Wortmannin*, or WTM, an inhibitor of the PI3K, Cytochalasin D, or CYTO D, an inhibitor of actin polymerization) autophagy, disrupt the secretory pathway (Brefeldin A; BFA), de novo protein synthesis (Cycloheximide; CHX) or the endo-lysosome pathway (Bafilomycin A, or Baf A1, an inhibitor of vacuolar type-H+ ATPase and Chloroquine or CHQ, both inhibiting the endosome acidification). MR1 surface expression was monitored by flow cytometry (26.5 clone; Fig. 5*A*) following overnight culture of U266 cells with the pharmacological inhibitors described above, paralleled by our functional assay testing the capacity of treated U266 to activate _conv_MAIT TCR-expressing Jurkat cells (Fig. 5*B*).

Although MR1 surface expression was barely detectable by flow cytometric analysis of U266 cells cultured in the presence of 5-OP-RU using the 26.5 antibody clone (Fig. 5*A*), pharmacological inhibitors including CHQ, Baf A1, BFA, and CHX significantly reduced MAIT TCR activation (Fig. 5*B*). Importantly, we further showed that while increasing MR1 expression detected by 26.5 antibody (Fig. 5*A*) and MAIT TCR MD01-005 J33-4 activation (Fig. 5 *B* and *C*), *E. faecalis* mitigated inhibition of MR1-dependent MAIT TCR activation by Baf A1 and CHQ (Fig. 5*B*). These data are consistent with the concept that *E. faecalis* may enhance 5-OP-RU-dependent surface MR1 expression through a posttranscriptional mechanism involving regulation of MR1 intracellular trafficking. Finally, although CYTO D and WTN did not alter MR1 cell surface expression upon 5-OP-RU/*E. faecalis* incubation, RAPA partially increased the expression of MR1 on cells cultured with 5-OP-RU (Fig. 5*A*) and MAIT TCR recognition (Fig. 5*C*), suggesting that autophagy may also contribute, at least partially, to the synergistic upregulation of MR1, consistent with the RNAseq data (Fig. 4). Because these pharmacological agents are not specific probes of individual trafficking steps, these experiments should be interpreted as pathway-informing rather than pathway-defining and the understanding of the precise compartments involved will require further investigation using genetic and imaging approaches.

### Concluding Remarks.

Our main finding is that select *Enterococcus* species can enhance cell-surface MR1 expression and thereby potentiate MR1-dependent activation of _conv_MAIT cells in the presence of exogenous ligand. Gut microbiota has emerged, in these past decades, as a potent actor in the efficacy of cancer treatment, especially immune checkpoint inhibitors (37, 38). However, little is known about the molecular mechanisms by which immunomodulatory microbes may act on cancer immunosurveillance and response to immunotherapy. We provide evidence consistent with a mechanism by which intratumoral bacteria may alter MR1 surface density on APCs, potentially enhancing the stimulation and expansion of innate-like T cells in the tumor microenvironment. Although riboflavin-auxotrophic *Enterococcus* spp. are not capable of directly stimulating MAIT cells, they synergistically enhanced the expression of ligand-loaded MR1 at the surface of APCs, including DCs, monocytic phagocytes, and B cells. These results suggest that certain intracellular pathogens may be sensed by the immune system through modification of MR1 density on the surface of infected cells.

The potential clinical relevance of the MR1–MAIT axis in cancer is supported by several lines of evidence, although prognostic and predictive associations should be distinguished. Direct evidence that tumor MR1 expression itself predicts response to checkpoint inhibitor therapy remains limited. However, MR1 expression has been associated with clinical outcome in some settings; notably, a TCGA-based analysis reported that MR1 overexpression correlated with poorer overall survival in glioma, indicating that MR1 expression may have prognostic value in at least some tumor types (39). In parallel, studies of the downstream immune compartment have linked MAIT-cell abundance or phenotype to anti-PD-1 response, including circulating MAIT cells in metastatic melanoma (40) and CXCR6+ CD8+ MAIT cells in NSCLC (20). Together, these observations support the broader clinical relevance of the MR1–MAIT pathway, though whether intratumoral MR1 expression itself predicts benefit from checkpoint blockade remains unresolved. Our observations may also be distinguished from the gut microbiota-dependent *Enterococcus* effects on checkpoint inhibitor efficacy reported by Griffin et al. (41). Whereas that work implicated bacterial peptidoglycan remodeling in systemic enhancement of antitumor immunity, our data instead support a local effect on MR1-dependent antigen presentation within the tumor microenvironment and mechanistically distinct from a generic innate adjuvant effect.

Although the present study was not designed to determine whether intratumoral *Enterococcus*, MR1 expression, or MAIT activation correlate with response to PD-1 blockade, these findings provide a mechanistic framework for future studies examining how tumor-associated bacteria influence MR1-dependent immune surveillance in cancer and potentially impact response to immunotherapy.

### Weaknesses of the Study.

Our mechanistic inferences rely largely on pharmacological perturbation. While these data support posttranscriptional regulation of surface MR1 expression, they do not establish the specific endosomal or lysosomal compartments involved. Future microscopy-based localization studies and genetic perturbation of trafficking regulators will be needed to resolve the mechanism. Whereas the scope of the study focuses on the role of *Enterococcus* in regulating MR1 expression for TCR recognition, we did not have the tissues available to isolate and characterize the intratumoral bacterial strains for their actual riboflavin auxotrophy. Although patient-derived datasets from a neoadjuvant anti-PD-1 setting provided the clinical context for identifying intratumoral MAIT clonotypes, the present data do not support conclusions regarding therapeutic benefit or detriment. The limited number of responding patients precluded correlative studies which could suggest the role of MAIT cells in pathological responses to PD-1 blockade. Finally, the study does not clarify whether *Enterococcal* species enter the target cells or remain extracellular to mediate their effects.

## Materials and Methods

### Lung Cancer Patient Single-Cell Dataset and MAIT Cell Cluster analysis.

Single-cell RNA and TCR sequencing data from patients with non–small cell lung cancer (NSCLC) treated with neoadjuvant ICB was obtained from previously published dataset (19). Sub-clustering of the annotated CD3+ MAIT cell cluster was performed using Seurat v5. Dimensional reduction was done using the RunUMAP function. Cell markers were identified by using a two-sided Wilcoxon rank sum test. Genes with adjusted *P* < 0.05 were retained. MAIT subclusters were annotated based on differential genes and immune cell markers. Top 20 differentially expressed genes for each cluster are shown in *SI Appendix*, Table S1. Conventional MAIT cells defined by the presence of TRAV1-2 gene were overlaid on MAIT cell population.

### Cell Lines and Reagents.

Genetically modified CD8-expressing Jurkat reporter cell line, CD8 + NFAT-lucΔαβ, was kindly provided by Kellie Smith (19). U266 cells were kindly provided by Ivan Borello’s lab and cultured in RPMI with 10% FBS, 200 μM glutamine, and 1% pen-strep. MeWo and SNU387 cell lines were kindly provided by Scott Wilson and cultured according to ATCC protocol. THP1 cells were obtained from ATCC and cultured following the provider’s recommendation. Beta-2 microglobulin-knockout (β2m KO) Jurkat cells and β2m KO U266 cells were generated via CRISPR-Cas9 system synthetic guide RNA (sgRNA) targeting: GAGTAGCGCGAGCACAGCTAAGG (Synthego, Redwood City, CA). An MR1-KO THP1 cell line was generated via CRISPR-Cas9 with sgRNA targeting: TGGAACTGAAGCGCCTACAGAGG.

5-A-RU-PABC-Val-Cit-Fmoc is a prodrug of the MR1 ligand 5-A-RU, a precursor of riboflavin (vitamin B2) and purchased at MedchemExpress (Monmouth Junction, NJ) and was used in experiments and described as 5-OP-RU. Brefeldin A and LPS were purchased from Thermofisher (Waltham, MA). Chloroquine was purchased from Biolegend (SanDiego, CA). Cycloheximide, Cytochalasin D, Bafilomycin A, Rapamycin, Wortmannin, and Lipoteichoic acid (LTA) were purchased from Millipore Sigma (Saint Louis, MI). TLR9 was purchased from Invivogen (SanDiego, CA).

### Bacteria.

Bacteria that were either able to produce riboflavin or deficient in production were cultured in medium as described in Table 1. Bacteria were aliquoted at defined colony-forming units (CFU). Clinical isolates were kindly provided by Cynthia Sears.

**Table 1.** Bacteria strains used in the TCR capture functional assay

Bacteria	Strain	Medium	
		Broth/plate	Oxygen
*E. coli*	DH5α	LB/LB	Aerobic
*K. pneumoniae*	Clinical isolate	BHI/BHI	Aerobic
*Enterococcus* spp. (*E. hirae, E. faecalis, E. faecium, E. avium*)	Clinical isolate	BHI/BHI	Aerobic
*Pseudomonas aeruginosa*	Clinical isolate	TSB/TSA	Aerobic
*Staphylococcus aureus*	SH1000	TSB/TSA blood	Aerobic
*S. epidermidis*	Clinical isolate	TSB/TSA	Aerobic
*B. thetaiotaomicron*	*VP5482*	BHI/BHI	Anaerobic
*B. longum*	ATCC15708	RC/BHI	Anaerobic

To prepare bacteria for use in MAIT cell assays, cultures were centrifuged at 4,000×*g* for 15 min. Bacterial pellets were resuspended in 50% glycerol:culture media and stored at −80 °C in single-use aliquot. Prior to use with mammalian cells in most assays, bacteria were fixed using 2% PFA for 15 min. Additional methods tested include freeze–thaw cycles (20 cycles liquid nitrogen/room temperature), heat inactivation (70 °C for 30 min), and sonication (*Q-SONICA* sonicator at 50 amplitude for 20 cycles of 10 s-sonication and 1 min-rest on ice). For live bacteria preincubation, experimental aliquots were washed three times with PBS and incubated with cell lines at 37 °C for different times, followed by vigorous wash with PBS (277×*g*, 5 min per time).

### TCR Transfection and Transient Expression.

The protocol was adapted from previously described methods (19). Briefly, we identified TRAV and TRBV families using the IMGT/V-Quest database (https://www.imgt.org) and the TCR were generated by fusing the TCRA V-J region sequences and the TCRB V-D-J regions with the human TRA and TRB constant chains, respectively. The full-length TCRA and TCRB chains were then produced as individual gene blocks and cloned into a pCI mammalian expression vector, containing a CMV promoter followed by transformation of competent NEB® 5-alpha Competent *E. coli (New England Biolabs)* following manufacturer protocol. The integrated plasmid DNA was extracted via QIAprep Spin Miniprep Kit and stored at −20 °C. DNA plasmid sequence was confirmed by Sanger sequencing (Genewiz). Jurkat reporter cells were then electroporated with pCI plasmid encoding TCRa and TCRb genes using ECM830 Square wave electroporation system (BTX) at 275 V for 10 ms in OptiMem media in a 4-mm cuvette. Postelectroporation, cells were rested overnight by incubating in RPMI 10% FBS at 37 °C at 5% CO_2_. TCR expression was confirmed by flow cytometric staining for CD3 expression.

### In Vitro MR1 Recognition Assay and Flow Cytometry.

1.5 × 10^5^ CD3+ TCR-expressing CD8 + NFAT-lucΔαβ reporter Jurkat cells were cocultured with 1.5 × 10^5^ target cells (WT THP1 and MR1 KO THP1) in the presence of prodrug 5-A-RU. Anti-CD3 (OKT3 clone) was used as a positive control. After overnight incubation, activation of the NFAT reporter gene was measured by the Bio-Glo Luciferase Assay following the manufacturer’s instructions (Promega). In some experiments APC were preincubated overnight with BFA (3 μg/mL), CHX (20 μg/mL), CHQ (100 μM), Bafilomycin A1 (100 nM), and Rapamycin (100 nM) in the presence of 6.25 nM of 5-OP-RU with *Enterococcus* spp. or vehicle control. Cells were then washed, and one aliquot was used to test MR1 surface expression by flow cytometry with anti-MR1 antibody 26.5 clone (2:100, Biolegend). The other aliquot was used to test TCR recognition as described above. TCR expression was confirmed by CD3 (2.5:100, OKT3, Biolegend) surface staining by flow cytometry. Briefly, cells were washed and stained with live/dead dye (near IR 1:1,000, Thermofisher) for 20 min in dark at room temperature followed by wash. Cells were then stained with surface stains described above for 30 min at 4 °C followed by wash.

### Bulk RNA Sequencing.

U266 myeloma cell line was cultured with or without (untreated control) riboflavin in the presence or absence of *E. coli* vs. *E. faecalis* overnight. RNA was extracted using MiniRNA kit (Qiagen; Germantown, MD), and bulk RNAseq was performed by Novogene (Sacramento, CA). The raw RNA-seq FASTQ files have been deposited on NCBI Gene Expression Omnibus (GEO) database and are accessible through accession number GSE320009.

### Human Lung Microbiome Sequencing.

FFPE tissues from patient tumor and normal lung were used to extract DNA with FFPE kit (Qiagen) and sent for whole genome and 16S rRNA sequencing (V3V4) to Novogene. Analysis was performed by Resphera Biosciences. For 16S, raw reads were processed using DADA2 filtering, DADA2 denoising, and chimera removal (42). Kraken2 and Metaphlan pipelines were used to analyze whole genome sequencing data by Resphera Biosciences (43, 44). Raw 16S rRNA amplicon sequencing FASTQ files have been deposited on the NCBI Sequence read archive (SRA) and are accessible through BioProject accession number PRJNA1425760.

### Metabolomic.

Bacteria cell pellets and culture supernatants of bacteria culture were analyzed for their content in riboflavin and folate using LC-MS approach by *Creative Proteomics* (Shirley, NY).

### ELISA Analysis.

IFNα, IL1β and TNFα were measured in the overnight U266 culture supernatants using R&D System ELISA kits following the manufacturer’s protocols.

### Statistics.

All statistical analyses were performed using Graphpad Prism v10 (excluding single RNA sequencing and bulk RNA sequencing analysis). A *P*-value of less than 0.05 was considered statistically significant. The unpaired two-tailed *t* test was used for statistical comparisons as indicated in the figure legends. *P* value legend is as follows: ****P < 0.0001, ***0.0001 < *P* < 0.001, **0.001 < *P* < 0.01, *0.01 < *P* < 0.05, ns (nonsignificant) by the unpaired *t* test (two-tailed).

## Supplementary Material

Appendix 01 (PDF)

## Data Availability

The code used for the analysis of the single-cell RNA sequencing and Bulk RNA sequencing have been deposited in GitHub at https://github.com/pbirla1/MAIT_NSCLC_enterococcus (45). The code used for the analysis of microbiome sequencing data have been made available on GitHub at https://github.com/pbirla1/jhmi-sears-pakhi-wgs-microbiome (46). Bulk RNA sequencing and microbiome 16S rRNA sequencing data have been deposited in GEO Accession No. GSE320009 (47) and SRA Accession No. PRJNA1425760 (48), respectively.
