# Supplementary material for: Select intratumoral riboflavin-auxotrophic Enterococcus species enhance cell surface MR1 expression and MAIT TCR activation in lung cancer
Source: Proc Natl Acad Sci U S A. 2026 Aug 18;123(34):e2617943123. doi: 10.1073/pnas.2617943123 (PMC13506043; doi:10.1073/pnas.2617943123)
Supplement: Supplementary file 1 [file pnas.2617943123.sapp.pdf]

PNAS

## Supporting Information for

# Select intratumoral riboflavin-auxotrophic Enterococcus species enhance MR1 surface expression and MAIT TCR activation in lung cancer

Pakhi Birla[1,2], Lansaol Yang[1,2], Wanting Shan[1,2], Sakura Minamisawa[3], Omkar Dhaygude[4], Haritha Manoj[1,2], Alex J. Lee[1,2], Jacqueline Ferri[1,2], Ying Zheng[1,2], Andrew Northcutt[1], Hongni Fan[1,2], Hadley Beauregard[5], Zhen Zeng[1,2], Kellie N. Smith[1,2], Fyza Y. Shaikh[1,2], Cynthia L. Sears[2,5], Drew M. Pardoll[1,2], Franck Housseau[1,2,6,7]

[1] Sidney Kimmel Comprehensive Cancer Center, Johns Hopkins University, Baltimore, MD, USA
[2] Bloomberg~Kimmel Institute for Cancer Immunotherapy, Johns Hopkins School of Medicine, Baltimore, MD, USA
[3] Department of Pathology and Oncology, Faculty of Medicine, Juntendo University, Tokyo, Japan
[4] Department of Mechanical Engineering, Whiting school of Engineering, Johns Hopkins University, Baltimore, MD, USA
[5] Department of Medicine, Division of Infectious Disease, Johns Hopkins University, Baltimore, MD, USA
[6] Current Address: CRCLille "Cancer Research Center Lille Laboratory" University of Lille-U1366 Inserm- CHU Lille-UMR9020 CNRS; Team Microbiote, Immunosurveillance of Infection and Cancer", room 4/14.36, ONCOLille bldg, Bd Professeur Jules Leclercq, 59000, Lille, France
[7] correspondence to: franck.housseau@inserm.fr

Corresponding author: Franck Housseau
Email: franck.housseau@inserm.fr

**This PDF file includes:**

Figures S1 to S3
Tables S1

## Supplementary Figures

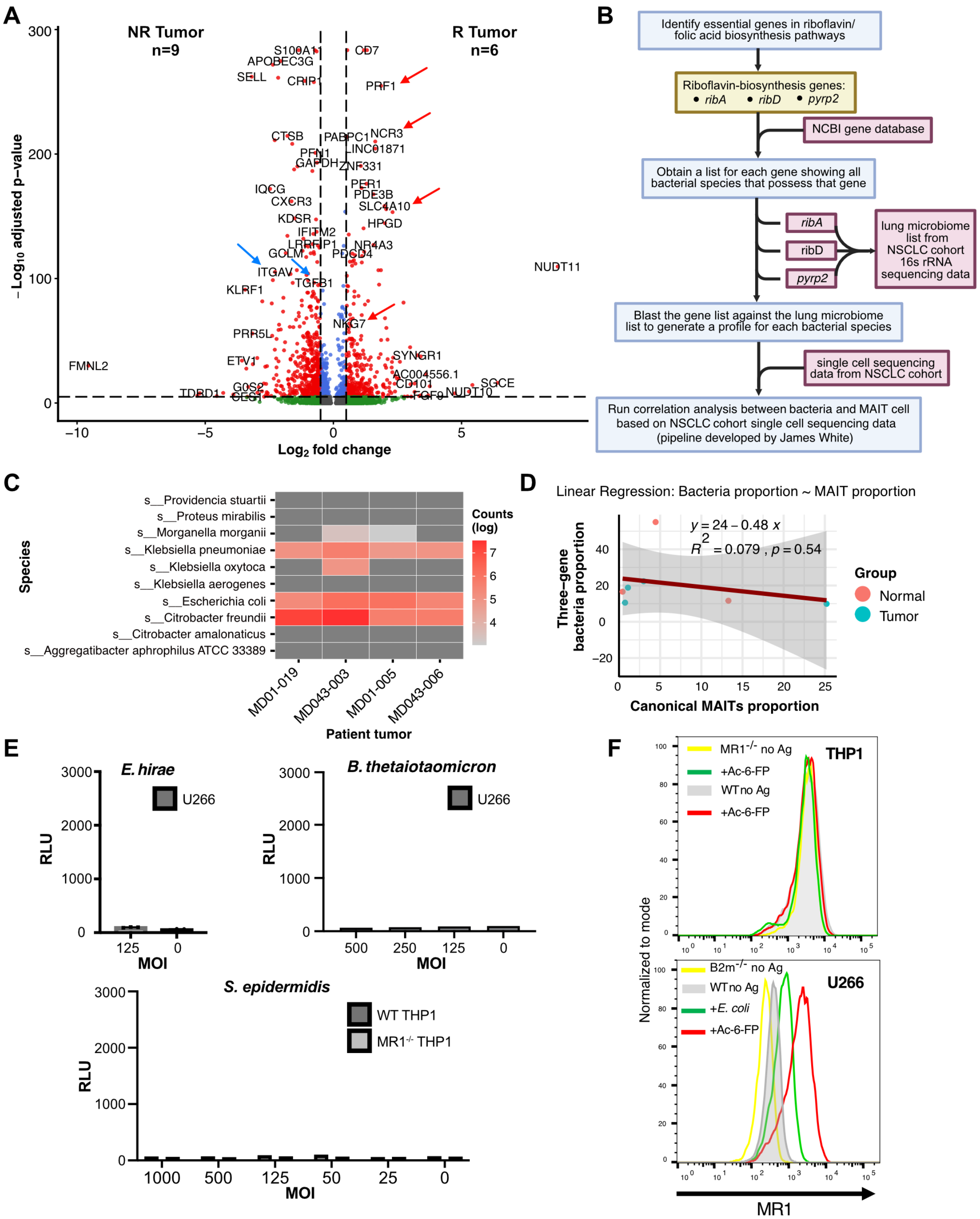


**Supplementary figure S1. A.** Volcano plot depicting differentially expressed genes in *cytotoxic MAIT 1* cluster in tumor tissues from responder (n = 6) vs non-responder (n = 9) patients. **B,** Schematic representation of the pipeline predicting the production of 5-A-RU by the intratumoral microbiota. **C**, Heat map representing the abundance of 5-A-RU producing bacteria among all bacteria in lung tumor samples. **D**, Linear regression analysis showing the absence of correlation between the proportion of intra-tumoral 5-A-RU-producing bacteria and proportion of intratumoral conventional MAIT cells (TCRAV1-2/J33,20,12+). **E**, Riboflavin auxotrophic *E. hirae*, riboflavin-

producing *B. thetaiotaomicron* and *S. epidermidis* do not activate MAIT TCR MD01-005 J33-4. **F**, Flow cytometric analysis of MR1 expression on WT THP1 (Top) and WT U266 (Bottom) without antigen or in the presence of *E. coli* or Ac-6-FP. MR1 negative $MR1^{-/-}$ THP1 and $b2m^{-/-}$ U266 are used as negative controls.

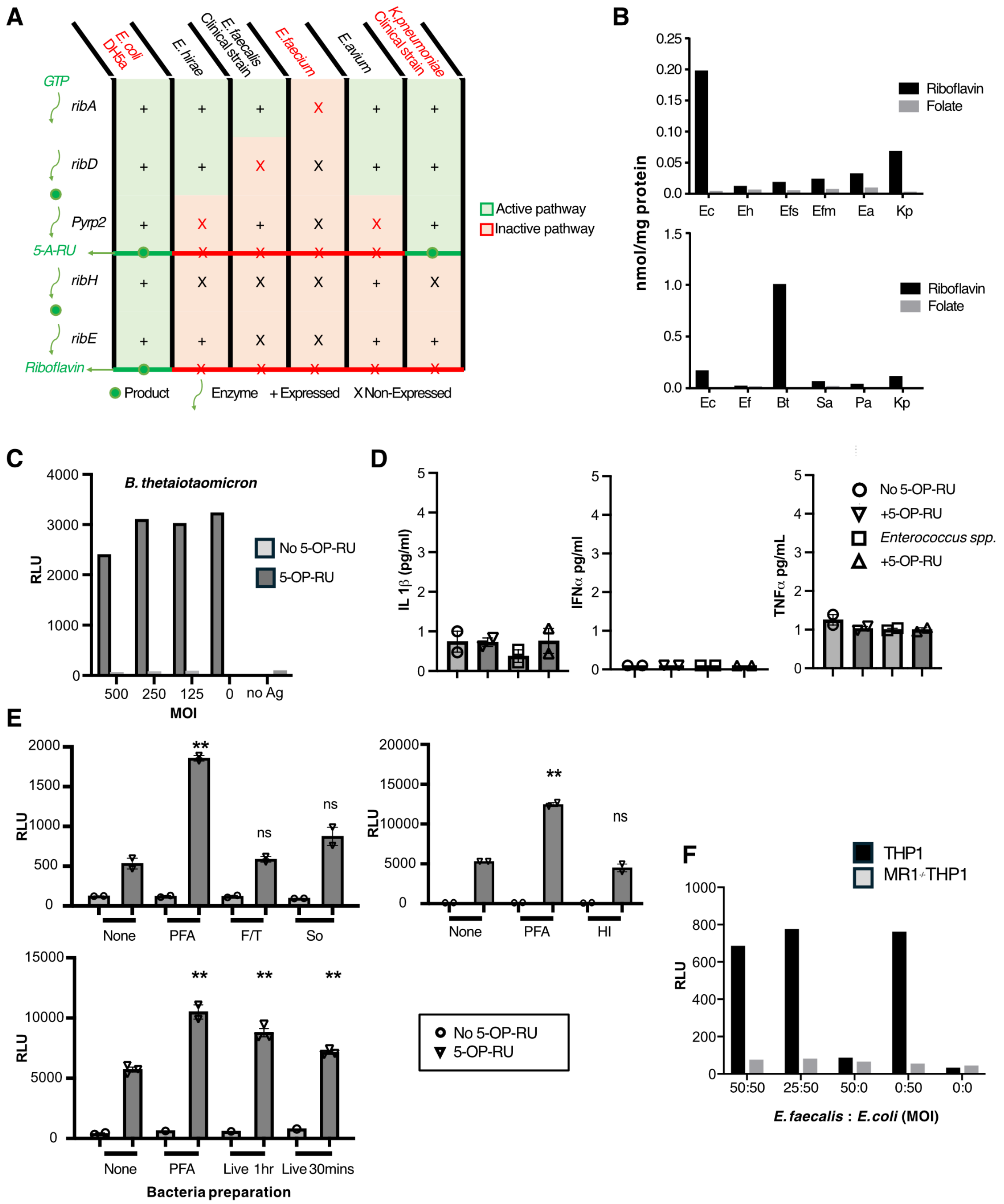


**Supplementary figure S2. A**, Bacteria strains used in our tests were sequenced to determine the expression of genes encoding the enzymes responsible for the biosynthesis of Riboflavin from GTP. +, presence of the gene; X, absence of the gene. Red boxes, deficient metabolic steps; Green, proficient metabolic steps. **B**, Metabolomic analysis of riboflavin biosynthesis by *E. coli* (Ec), *E. hirae* (Eh) *E. faecalis* (Efs), *E. faecium* (Efm), *E. avium* (Ea), *K. pneumoniae* (Kp), *B. thetaiotamicron* (Bt), *Staphylococcus aureus* (SA), *Pseudomonas aeruginosa* (PA) to validate the genomic analysis in **A. C**, MAIT TCR J33-4 recognition of U266 cells incubated overnight with (dark grey) or without (light grey) 5-OP-RU, in the presence or absence of decreasing MOI of *B. thetaiotaomicron*. TCR activation is measured by bioluminescence reading. **D**, IL1b, INFa, and TNFa measurements by ELISA, in the culture supernatants of U266 cultured with (inverse triangles)

or without (circles) 5-OP-RU, in the presence of *Enterococcus spp.* alone (square) or in combination with 5-OP-RU (triangles). **E**, MAIT TCR J33-4 recognition of U266 cells incubated overnight with (inverse triangles) or without (circles) 5-OP-RU, in the presence or absence of *E. coli*, PFA fixed (PFA), heat inactivated (HI), sonicated (So), Frozen/Thawed lysed (F/T) or live bacteria preparations. **F**, absence of synergy by *E. faecalis* on TCR J33-4 activation by THP1 cells cultured in the presence of PFA fixed *E. coli* at different MOIs. $MR1^{-/-}$ THP1 cells were used as negative control.

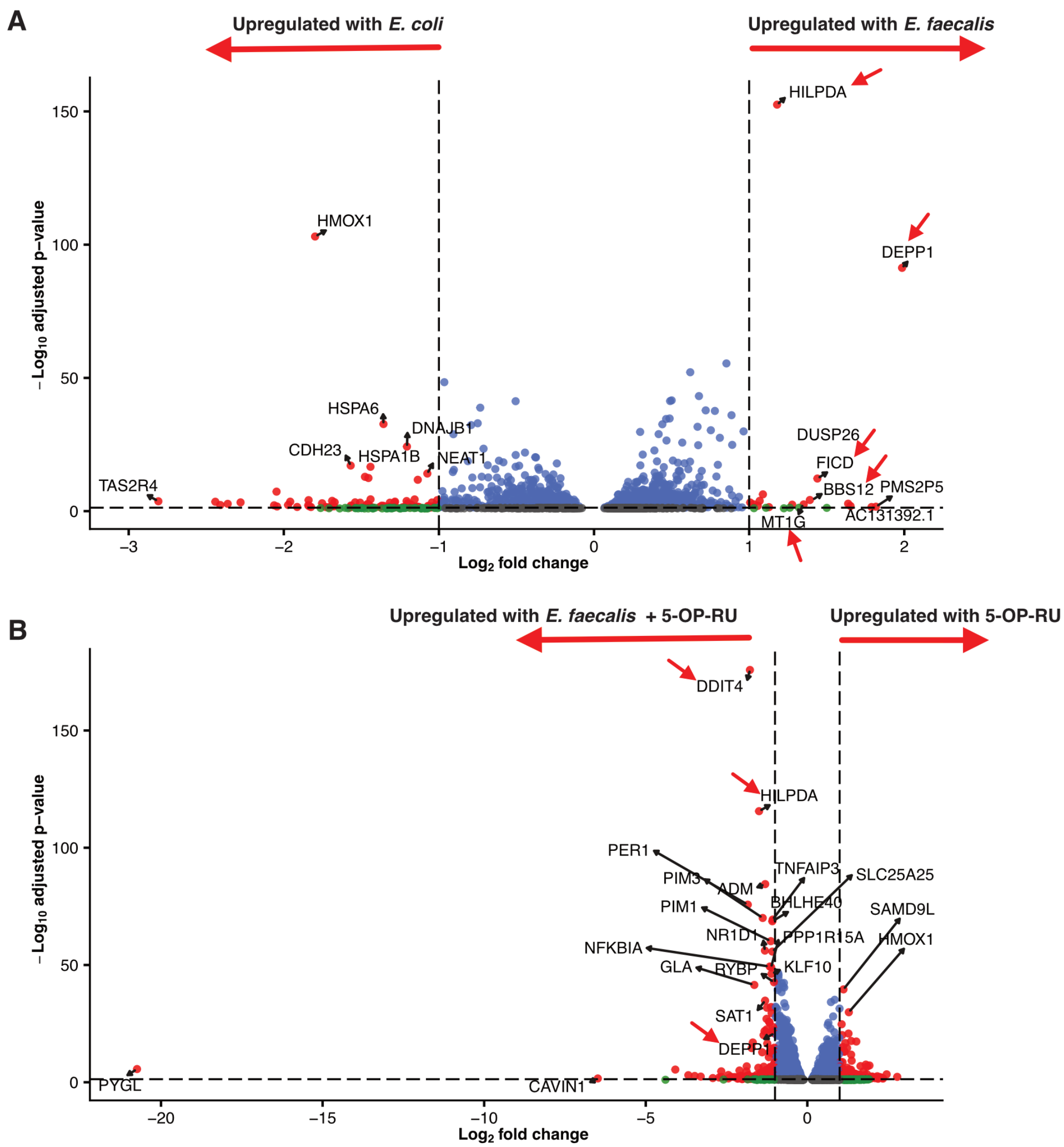


**Supplementary figure S3. A&B,** Volcano plots representing RNAseq analysis of U266 cells incubated with *E. faecalis* compared to *E. coli* (**A**) and 5-OP-RU + *E.* faecalis compared to 5-OP-RU alone (**B**). Significant genes were selected when adjusted p-value<0.01 and -Log2(FC)>1. Red arrows indicate genes associated with autophagy.

**Supplementary Table S1.** Table showing top 20 differentially expressed genes by each MAIT sub-cluster depicted in Fig. 1A.

| | p_val | avg_log2FC | pct.1 | pct.2 | p_val_adj | cluster | gene |
|---|---|---|---|---|---|---|---|
| 1 | 0 | 1.77264673 | 0.644 | 0.189 | 0 | Cytotoxic MAIT 1 | GZMK |
| 2 | 0 | 1.215691413 | 0.663 | 0.316 | 0 | Cytotoxic MAIT 1 | GZMA |
| 3 | 0 | 1.24158022 | 0.753 | 0.441 | 0 | Cytotoxic MAIT 1 | CST7 |
| 4 | 0 | 1.059451844 | 0.552 | 0.261 | 0 | Cytotoxic MAIT 1 | CTSW |
| 5 | 0 | 1.098115008 | 0.463 | 0.188 | 0 | Cytotoxic MAIT 1 | NKG7 |
| 6 | 0 | 1.106422151 | 0.658 | 0.405 | 0 | Cytotoxic MAIT 1 | SLC7A5 |
| 7 | 0 | 1.443754399 | 0.469 | 0.217 | 0 | Cytotoxic MAIT 1 | GYG1 |
| 8 | 0 | 0.96428003 | 0.718 | 0.483 | 0 | Cytotoxic MAIT 1 | LITAF |
| 9 | 0 | 1.16999167 | 0.43 | 0.2 | 0 | Cytotoxic MAIT 1 | CCL4 |
| 10 | 0 | 1.049270398 | 0.536 | 0.317 | 0 | Cytotoxic MAIT 1 | IFNGR1 |
| 11 | 0 | 1.612693343 | 0.349 | 0.141 | 0 | Cytotoxic MAIT 1 | PRF1 |
| 12 | 0 | 2.07753191 | 0.268 | 0.08 | 0 | Cytotoxic MAIT 1 | NCR3 |
| 13 | 0 | 1.642682726 | 0.312 | 0.132 | 0 | Cytotoxic MAIT 1 | APOBEC3G |
| 14 | 0 | 1.119553914 | 0.303 | 0.14 | 0 | Cytotoxic MAIT 1 | KLRG1 |
| 15 | 0 | 1.291246619 | 0.274 | 0.121 | 0 | Cytotoxic MAIT 1 | MATK |
| 16 | 0 | 1.572221552 | 0.259 | 0.111 | 0 | Cytotoxic MAIT 1 | OASL |
| 17 | 1.03E-228 | 1.354742737 | 0.253 | 0.126 | 2.30E-224 | Cytotoxic MAIT 1 | AC245014.3 |
| 18 | 3.82E-216 | 1.045400663 | 0.322 | 0.188 | 8.48E-212 | Cytotoxic MAIT 1 | EFHD2 |
| 19 | 3.71E-212 | 0.84251088 | 0.344 | 0.2 | 8.25E-208 | Cytotoxic MAIT 1 | LYAR |
| 20 | 2.48E-203 | 0.90430553 | 0.327 | 0.19 | 5.50E-199 | Cytotoxic MAIT 1 | IL4I1 |
| 21 | 0 | 2.074835152 | 0.556 | 0.207 | 0 | Naïve MAIT 1 | CCR7 |
| 22 | 9.86E-276 | 0.686261855 | 0.715 | 0.611 | 2.19E-271 | Naïve MAIT 1 | LDHB |
| 23 | 5.55E-263 | 0.956900289 | 0.519 | 0.369 | 1.23E-258 | Naïve MAIT 1 | ITGB1 |
| 24 | 8.28E-260 | 0.686224805 | 0.788 | 0.698 | 1.84E-255 | Naïve MAIT 1 | LTB |
| 25 | 8.65E-254 | 0.754689209 | 0.683 | 0.557 | 1.92E-249 | Naïve MAIT 1 | GPR183 |
| 26 | 2.55E-247 | 1.337476101 | 0.293 | 0.149 | 5.67E-243 | Naïve MAIT 1 | TNFRSF4 |
| 27 | 4.32E-245 | 1.442645069 | 0.268 | 0.131 | 9.61E-241 | Naïve MAIT 1 | ICAM2 |
| 28 | 2.06E-207 | 0.60360765 | 0.703 | 0.602 | 4.57E-203 | Naïve MAIT 1 | EMP3 |
| 29 | 4.17E-182 | 1.488058706 | 0.27 | 0.153 | 9.28E-178 | Naïve MAIT 1 | SELL |
| 30 | 1.80E-130 | 0.912050689 | 0.378 | 0.271 | 4.00E-126 | Naïve MAIT 1 | GIMAP7 |
| 31 | 9.71E-129 | 0.629531005 | 0.537 | 0.444 | 2.16E-124 | Naïve MAIT 1 | RNASET2 |
| 32 | 5.10E-122 | 0.889377259 | 0.324 | 0.22 | 1.13E-117 | Naïve MAIT 1 | TIMP1 |
| 33 | 4.27E-112 | 0.735359161 | 0.418 | 0.32 | 9.49E-108 | Naïve MAIT 1 | ITK |
| 34 | 6.10E-87 | 0.729429615 | 0.351 | 0.264 | 1.35E-82 | Naïve MAIT 1 | KLF3 |
| 35 | 1.85E-86 | 0.61164141 | 0.423 | 0.34 | 4.10E-82 | Naïve MAIT 1 | ARHGAP15 |
| 36 | 8.14E-70 | 0.759144246 | 0.267 | 0.195 | 1.81E-65 | Naïve MAIT 1 | INPP4B |
| 37 | 5.60E-69 | 0.59896484 | 0.371 | 0.297 | 1.24E-64 | Naïve MAIT 1 | FLT3LG |
| 38 | 1.58E-59 | 0.778228044 | 0.254 | 0.188 | 3.51E-55 | Naïve MAIT 1 | ARRDC2 |
| 39 | 3.30E-59 | 0.7184492 | 0.254 | 0.187 | 7.34E-55 | Naïve MAIT 1 | ADD3 |
| 40 | 3.45E-34 | 0.606966844 | 0.251 | 0.203 | 7.67E-30 | Naïve MAIT 1 | SAMHD1 |
| 41 | 0 | 1.21554164 | 0.75 | 0.404 | 0 | MAIT 17 | RGS1 |
| 42 | 0 | 1.70216016 | 0.505 | 0.191 | 0 | MAIT 17 | MAF |
| 43 | 0 | 1.285366816 | 0.585 | 0.271 | 0 | MAIT 17 | ARID5B |
| 44 | 0 | 1.990411658 | 0.431 | 0.118 | 0 | MAIT 17 | CTLA4 |
| 45 | 0 | 1.638846194 | 0.558 | 0.265 | 0 | MAIT 17 | CXCR6 |
| 46 | 0 | 1.967685617 | 0.398 | 0.112 | 0 | MAIT 17 | PTPN13 |
| 47 | 0 | 1.340305246 | 0.475 | 0.205 | 0 | MAIT 17 | CD4 |

| | | | | | | | |
|---|---|---|---|---|---|---|---|
| 48 | 0 | 1.205118943 | 0.517 | 0.257 | 0 | MAIT 17 | ICOS |
| 49 | 0 | 1.677033028 | 0.372 | 0.117 | 0 | MAIT 17 | SH3TC1 |
| 50 | 0 | 1.75996129 | 0.359 | 0.107 | 0 | MAIT 17 | CCR4 |
| 51 | 0 | 1.374695036 | 0.441 | 0.193 | 0 | MAIT 17 | TMEM173 |
| 52 | 0 | 2.144327859 | 0.336 | 0.096 | 0 | MAIT 17 | MIR4435-2HG |
| 53 | 0 | 1.284899299 | 0.335 | 0.144 | 0 | MAIT 17 | ZEB2 |
| 54 | 4.30E-289 | 1.08912275 | 0.397 | 0.203 | 9.55E-285 | MAIT 17 | IKZF3 |
| 55 | 3.39E-275 | 1.209332349 | 0.317 | 0.144 | 7.54E-271 | MAIT 17 | PHTF2 |
| 56 | 4.49E-268 | 1.238407228 | 0.306 | 0.139 | 9.98E-264 | MAIT 17 | PDCD1 |
| 57 | 2.59E-248 | 1.034547847 | 0.354 | 0.179 | 5.76E-244 | MAIT 17 | LIMS1 |
| 58 | 1.71E-246 | 1.107198504 | 0.343 | 0.171 | 3.81E-242 | MAIT 17 | FURIN |
| 59 | 1.15E-216 | 0.969830493 | 0.451 | 0.275 | 2.57E-212 | MAIT 17 | BATF |
| 60 | 4.95E-180 | 0.986586648 | 0.319 | 0.175 | 1.10E-175 | MAIT 17 | ZBTB38 |
| 61 | 0 | 2.585912013 | 0.858 | 0.256 | 0 | Activated MAIT 1 | NKG7 |
| 62 | 0 | 1.650699812 | 0.842 | 0.328 | 0 | Activated MAIT 1 | GZMK |
| 63 | 0 | 2.004988227 | 0.717 | 0.259 | 0 | Activated MAIT 1 | CCL4 |
| 64 | 0 | 1.961078568 | 0.985 | 0.542 | 0 | Activated MAIT 1 | CCL5 |
| 65 | 0 | 1.568641893 | 0.775 | 0.344 | 0 | Activated MAIT 1 | CTSW |
| 66 | 0 | 3.962873545 | 0.44 | 0.035 | 0 | Activated MAIT 1 | KLRD1 |
| 67 | 0 | 1.474905598 | 0.925 | 0.534 | 0 | Activated MAIT 1 | CST7 |
| 68 | 0 | 1.581090191 | 0.584 | 0.196 | 0 | Activated MAIT 1 | PRF1 |
| 69 | 0 | 3.540656174 | 0.386 | 0.048 | 0 | Activated MAIT 1 | GZMB |
| 70 | 0 | 1.701967406 | 0.466 | 0.13 | 0 | Activated MAIT 1 | CD8A |
| 71 | 0 | 1.886538853 | 0.474 | 0.157 | 0 | Activated MAIT 1 | HLA-DRB1 |
| 72 | 0 | 2.32489915 | 0.302 | 0.06 | 0 | Activated MAIT 1 | GZMH |
| 73 | 0 | 3.188813373 | 0.264 | 0.031 | 0 | Activated MAIT 1 | CRTAM |
| 74 | 0 | 2.249669285 | 0.283 | 0.056 | 0 | Activated MAIT 1 | CD8B |
| 75 | 3.06E-279 | 1.664455419 | 0.465 | 0.16 | 6.80E-275 | Activated MAIT 1 | HLA-DPB1 |
| 76 | 5.56E-276 | 1.674326933 | 0.398 | 0.119 | 1.24E-271 | Activated MAIT 1 | AOAH |
| 77 | 7.45E-225 | 1.445513028 | 0.52 | 0.22 | 1.65E-220 | Activated MAIT 1 | HLA-DPA1 |
| 78 | 1.47E-206 | 1.779159161 | 0.295 | 0.087 | 3.26E-202 | Activated MAIT 1 | SLA2 |
| 79 | 4.66E-159 | 1.734167713 | 0.253 | 0.079 | 1.04E-154 | Activated MAIT 1 | HLA-DQB1 |
| 80 | 5.31E-158 | 1.628486619 | 0.328 | 0.123 | 1.18E-153 | Activated MAIT 1 | CMC1 |
| 81 | 0 | 1.829866985 | 0.899 | 0.484 | 0 | TRM | TXNIP |
| 82 | 1.21E-238 | 1.95146465 | 0.513 | 0.188 | 2.68E-234 | TRM | KLRG1 |
| 83 | 1.88E-181 | 1.337268493 | 0.668 | 0.345 | 4.17E-177 | TRM | GZMK |
| 84 | 6.77E-173 | 1.313375322 | 0.912 | 0.831 | 1.50E-168 | TRM | CD69 |
| 85 | 8.05E-157 | 2.123059927 | 0.345 | 0.119 | 1.79E-152 | TRM | ARRDC3 |
| 86 | 1.97E-138 | 1.833961904 | 0.351 | 0.127 | 4.38E-134 | TRM | ADRB2 |
| 87 | 1.10E-123 | 1.69955112 | 0.382 | 0.157 | 2.45E-119 | TRM | AL118516.1 |
| 88 | 2.40E-121 | 1.724814666 | 0.316 | 0.115 | 5.33E-117 | TRM | LINC00861 |
| 89 | 1.95E-120 | 1.712141264 | 0.332 | 0.127 | 4.34E-116 | TRM | CMC1 |
| 90 | 2.85E-110 | 1.616299984 | 0.293 | 0.107 | 6.33E-106 | TRM | IL18RAP |
| 91 | 4.02E-107 | 1.411721207 | 0.513 | 0.276 | 8.94E-103 | TRM | CCL4 |
| 92 | 6.02E-104 | 1.854853357 | 0.306 | 0.12 | 1.34E-99 | TRM | RBKS |
| 93 | 9.61E-104 | 1.323629668 | 0.436 | 0.208 | 2.14E-99 | TRM | GBP5 |
| 94 | 3.27E-101 | 1.572052922 | 0.371 | 0.164 | 7.26E-97 | TRM | CD83 |
| 95 | 3.08E-95 | 1.608951772 | 0.273 | 0.104 | 6.85E-91 | TRM | ILF3-DT |
| 96 | 1.47E-88 | 1.465378183 | 0.334 | 0.146 | 3.28E-84 | TRM | AC243960.1 |
| 97 | 6.76E-86 | 1.602165804 | 0.381 | 0.194 | 1.50E-81 | TRM | AC004687.1 |

| | | | | | | | |
|---|---|---|---|---|---|---|---|
| 98 | 3.58E-82 | 1.400440304 | 0.3 | 0.128 | 7.95E-78 | TRM | DENND2D |
| 99 | 4.22E-78 | 1.743268565 | 0.263 | 0.108 | 9.37E-74 | TRM | DUSP6 |
| 100 | 2.06E-62 | 1.240296852 | 0.354 | 0.188 | 4.57E-58 | TRM | ATP2B1-AS1 |
| 101 | 0 | 3.558818439 | 0.914 | 0.121 | 0 | Activated tissue resident | CD8A |
| 102 | 0 | 4.270999163 | 0.678 | 0.047 | 0 | Activated tissue resident | CD8B |
| 103 | 0 | 2.877887528 | 0.543 | 0.093 | 0 | Activated tissue resident | SPRY1 |
| 104 | 0 | 2.416229393 | 0.578 | 0.15 | 0 | Activated tissue resident | ITGAE |
| 105 | 0 | 1.431751658 | 0.951 | 0.552 | 0 | Activated tissue resident | CCL5 |
| 106 | 0 | 4.779498229 | 0.4 | 0.019 | 0 | Activated tissue resident | LINC02446 |
| 107 | 0 | 3.293311355 | 0.417 | 0.054 | 0 | Activated tissue resident | CHN1 |
| 108 | 0 | 2.773031057 | 0.369 | 0.047 | 0 | Activated tissue resident | KLRD1 |
| 109 | 0 | 3.911411232 | 0.305 | 0.023 | 0 | Activated tissue resident | KLRC1 |
| 110 | 2.22E-275 | 2.058504635 | 0.46 | 0.124 | 4.93E-271 | Activated tissue resident | TMIGD2 |
| 111 | 3.23E-216 | 1.545546513 | 0.667 | 0.29 | 7.17E-212 | Activated tissue resident | LINC01871 |
| 112 | 3.80E-174 | 1.689118458 | 0.471 | 0.175 | 8.45E-170 | Activated tissue resident | ZEB2 |
| 113 | 3.08E-162 | 1.224833685 | 0.737 | 0.41 | 6.84E-158 | Activated tissue resident | GABARAPL1 |
| 114 | 1.56E-153 | 1.566335337 | 0.546 | 0.253 | 3.47E-149 | Activated tissue resident | LDLRAD4 |
| 115 | 5.22E-136 | 1.602335414 | 0.362 | 0.127 | 1.16E-131 | Activated tissue resident | AOAH |
| 116 | 9.93E-119 | 1.156563477 | 0.63 | 0.354 | 2.21E-114 | Activated tissue resident | PTPN22 |
| 117 | 6.24E-108 | 1.416182389 | 0.393 | 0.164 | 1.39E-103 | Activated tissue resident | FABP5 |
| 118 | 6.22E-91 | 1.159980116 | 0.505 | 0.265 | 1.38E-86 | Activated tissue resident | GLUL |
| 119 | 3.27E-51 | 1.142962018 | 0.272 | 0.129 | 7.26E-47 | Activated tissue resident | HPGD |
| 120 | 5.65E-47 | 1.362378938 | 0.314 | 0.17 | 1.26E-42 | Activated tissue resident | HLA-DRB1 |
